# Life Events and Coping Strategies Among Young People Who Died by Suicide or Sudden Violent Death

**DOI:** 10.3389/fpsyt.2021.670246

**Published:** 2021-08-27

**Authors:** Annelie Werbart Törnblom, Kimmo Sorjonen, Bo Runeson, Per-Anders Rydelius

**Affiliations:** ^1^Department of Women's and Children's Health, Centre for Psychiatry Research, Karolinska Institutet, Stockholm County Council, Stockholm, Sweden; ^2^Division of Psychology, Department of Clinical Neuroscience, Karolinska Institutet, Stockholm, Sweden; ^3^Department of Clinical Neuroscience, Centre for Psychiatry Research, Karolinska Institutet, Stockholm County Council, Stockholm, Sweden

**Keywords:** suicide, sudden violent death, adverse childhood experiences, stressful life events, coping strategies, children, adolescents and young adults

## Abstract

**Objective:** Most empirically anchored psychological models of suicide focus either on the perceived situational stress or on vulnerability factors and coping deficits. The interaction between life stressors and vulnerability factors is less explored.

**Methods:** This case-control study examines interactions between life events and coping strategies in three groups of young people: cases of suicide, cases of other sudden violent death (SVD), and control cases.

**Results:** Four coping strategies, two more adaptive and two more maladaptive, were identified. Distinctive of the suicide and the SVD group was significantly less Planful Problem-Solving, and more Escape-Avoidance and Confrontive Coping than among the controls. Furthermore, Confrontive Coping had significantly higher level in the SVD group than in the suicide group. Between-group differences were partly accounted for differences in negative life events, early and late in life. Both target groups experienced significantly more adverse childhood experiences and recent stressful life events than the controls—the suicide group being more exposed to recent stressful life events even in comparison with the SVD group. This might indicate that adverse childhood experiences are a risk factor for both causes of death, whereas proximal stressful life events are a risk factor for death by suicide to a higher degree than for SVD.

**Conclusions:** Improved understanding of the interplay between life events, both in the far past and present, and coping styles, may facilitate the identification of young people at risk of suicide and violent death.

## Introduction

Nowadays, we have solid, empirically supported knowledge of risk factors for death by suicide among adolescents and young adults, often generated by psychological autopsy studies (1–6). Most empirically anchored psychological models of suicide focus either on the perceived situational stress or on vulnerability factors and coping deficits (7). The interaction between life stressors and vulnerability factors, even if theoretically acknowledged, has been less explored (8). According to Joiner's interpersonal theory of suicide (9, 10), *thwarted belongingness* (feeling of disconnection from others) and *perceived burdensomeness* (being a burden to others), together with the acquired ability to enact lethal self-injury in response to repeated exposure to physically painful and/or fear-inducing experiences, are pre-requisites for suicide.

Previous research has demonstrated that individuals who make suicide attempts or have suicidal ideation, in comparison with non-suicidal persons, use less adaptive coping styles (11–14), see fewer solutions to their problems (15), expect more negative consequences of potential solutions (16), and are more likely to use avoidance and less active strategies (17). However, this might be state-dependent as a consequence of negative life stress or depression rather than a trait (7). High life stress can lead to a reduction in the use of adaptive coping in suicidal adolescents (14). Deficits in interpersonal problem-solving increase the risk of suicidal behavior as a reaction to stress (16). Individuals who perceive their attempts at solving problems to be unsuccessful might feel entrapped and powerless to escape from the situation (18, 19). A research review (20) found effective problem-solving to be a protective factor against suicide. Taken together, these studies indicate a need for further research on stressful life events as mediators and coping strategies as dependent variables.

Numerous studies have shown that individuals who died by suicide had significantly more adverse childhood experiences and had experienced more stressful life events the previous year, as compared to non-suicidal individuals. Childhood adversity is a risk factor for death by suicide (21–25) as well as for risk-taking behavior (26) and justice involvement (27). Both suicidal ideation and delinquency have common antecedents, namely impulsivity and aggression, substance misuse, and family adversity (28, 29). During adolescence and emerging adulthood, the risks of suicidal ideation and attempts increase dramatically as the number of adverse life events increases (30, 31). Adolescents who died by suicide were more likely to have experienced recent conflict with parents or with boy/girlfriends, disruption of a romantic attachment, and legal or disciplinary problems (32). Children and adolescents (aged 10–16) who died by suicide had significantly higher odds ratios for having experienced a recent painful loss (such as the loss of a family member, friend or other significant relation, a pet or other animal) or stressful conflict (with peers, school, parents or police), compared to accident victims (33, 34). Reviews of empirical studies have shown that recent adverse life events, particularly interpersonal stressors, represent a risk factor for severe forms of suicidal ideation and behavior, as well as death by suicide (35, 36). Even if these reviews suggest that such effects are partially independent of mental disorders, other authors (37) emphasize that “life stressors may serve as triggers to a vulnerable youth who may already be at risk” (p. 109).

However, we know much less about the risk factors for other forms of unnatural sudden death, such as accidents, homicide, or deaths from undetermined causes. Sudden violent death might be “latent suicides” or consequences of destructive and self-destructive behavior (38–42). Furthermore, we still need to develop empirically anchored models addressing the interplay among these multiple factors. One such attempt is the two-stage model of suicide and violence (7), based on the assumption that both are expressions of the same underlying aggressive impulse, and that other intervening variables determine whether the aggression is directed toward others or toward oneself (43–45). One such intervening variable is coping. Psychiatric inpatients admitted to a psychiatric unit following a suicide attempt scored higher on both suicide risk and violence risk, and both risks were predicted by different coping styles: suicide risk was significantly and negatively correlated with minimization, replacement and blame, whereas violence risk was positively correlated with blame (11).

To sum up, the number of potential risk factors is large, the relationships between them are complicated, and in each case we encounter unique, idiosyncratic combinations of fixed and variable, proximal, distal, and mediating variables (20, 46). In a previous study, we examined common and specific risk factors for dying young from suicide and other forms of sudden violent death (47). We found that borderline personality disorder was associated with both causes of death; depression spectrum disorder was associated with death by suicide, whereas antisocial personality disorder was associated with sudden violent death. Being investigated or sentenced for criminal acts was more common among cases of sudden violent death than among the controls. Noticeably, being bullied was negatively associated with belonging to the sudden violent death group rather than the control group, whereas being sexually assaulted was positively associated with belonging to the suicide group rather than the control group. Living in a steady relationship seemed to be a protective factor against suicide, whereas being a man was a risk factor for sudden violent death. The present study focuses on two specific aspects of the potential risk factors and their interplay: life events, both in the far past (adverse childhood experiences) and preceding the death (recent life stressors), and coping styles. These factors that are the focus of the present study are active in each individual case of suicide and sudden violent death, and might have an independent impact, even after controlling for psychopathology (20, 35, 36).

The overarching aim was to examine associations between life events, early and late in life, and coping strategies in three groups of young people, corresponding to: cases of suicide, other forms of sudden violent death, and control cases. Are possible differences in coping strategies between these groups to some degree accounted for (i.e., mediated) by differences in life events? Are there gender differences in this respect? Furthermore, we study the role of age in adverse childhood experiences, recent stressful life events, and coping strategies. Based on the previous findings described above, we predict less adaptive coping among young people who commit suicide or die of other types of sudden violent deaths. Furthermore, we expect these differences in coping to be accounted for, to some degree, by differences in childhood adversity or more recent stressful life events.

## Materials and Methods

### Data Collection

To meet the objective of this study, the case-control design was regarded as the method of choice. The total sample consisted of 229 subjects (children, adolescents, and young adults up to the age of 25 years). The sample included 63 prospectively collected consecutive cases of suicide and 62 consecutive cases of other forms of sudden violent death (SVD; murder, accident, unclear accident), identified at the Department of Forensic Medicine in Stockholm, Sweden, during a period of 4 years and 4 months (84 and 82% of all cases, respectively). Information on cause of death was based on autopsy protocols and police reports. About 3 months post-mortem, family members were contacted by letter and then by telephone. At least one interview per case was conducted (range 1–4), preferably with parents, with 105 interviews in the suicide group (one interview in 27 cases and multiple informants in 36 cases) and 91 interviews in the sudden violent death group (one interview in 35 cases and multiple informants in 27 cases). Furthermore, the sample included 104 living matched control cases, collected from the population registry in Stockholm County, using a randomized sample matched on age and gender. The 240 interviews in this group included 104 interviews with the young person, besides 136 parental interviews (one interview in one case and multiple informants in 103 cases). The responders in the control group, both the young people and their next-of-kin, were informed that they were a comparison group in a study of suicide and other forms of sudden violent death among children, adolescents, and young adults in the same age group as the young person.

The first author conducted the 436 tape-recorded interviews, lasting 3–4 h, separately with each participant at home. The interviews followed the psychological autopsy procedure (4, 5, 39, 48, 49). We used multiple informants, when possible, for each case included in the analyses. For each quantitative interview item, the answers were weighed by the first author to obtain best estimates and to cover the maximum of relevant information. Thus, the analysis units in the present study were the 229 cases in the three groups, and not the 436 interviews.

### Participants

Of the 63 cases of suicide, 41 were men (65.1%) and 22 were women (34.9%), aged 12–25 years (*M* = 20.9; *SD* = 3.0). Seven of the cases of suicide (11%) were younger than 18 years. Among the 62 cases of SVD, 55 were men (88.7%) and seven women (11.3%), aged 10–25 years (*M* = 20.6; *SD* = 3.5). Ten of the cases of SVD (16%) were younger than 18 years. Among the 104 control cases, 76 were men (73.1%) and 28 women (26.9%), aged 10–25 years (*M* = 20.7; *SD* = 3.4). Detailed sociodemographic, psychosocial and psychiatric data for the three samples, as well as previous suicide attempts, suicide methods and cause of sudden violent death are presented in a previous publication (47). However, it is worth noticing here that 71% of the cases of suicide, 58% of the cases of SVD, as well as 47% of the controls had received some form of help from mental health services.

### Measures

The protocol included a series of questions with set responses and open-ended questions about both early adversities in life and stressful life events in the previous year. As important information could appear not only as direct answers to these questions but often also in other parts in the interview, the scores on the three measures were assessed using all relevant interview information. The interview answers were transformed into scores for the three measures of stressful life events by two trained judges striving for consensus ratings.

The following measures were calculated for each subject, based on the items included in the interviews: Adverse Childhood Experiences (ACE), Social Readjustment Index (SRI), Life Event Index (LEI), and factor scores on the four factors obtained from factor analysis of Shortened Ways of Coping Questionnaire (WCQ). Together, these instruments covered both early and late adversities in life, as well as coping strategies when confronted with various forms of life stress.

Adverse Childhood Experiences (ACEs) is a concept from the CDC-Kaiser ACE Study (50), originally reported in 1998 (51) and showing overwhelming evidence for a lasting impact of early adversity. For example, a person with an ACE score ≥4 (out of 10) is 460% more likely to be depressed and 1,220% more likely to attempt suicide than a person with an ACE score of 0 (52). The 10 ACEs measured in the present study are identical to those applied in most recent ACE studies (53): abuse variables (emotional and verbal abuse, physical abuse, sexual abuse), neglect variables (emotional neglect, physical neglect), and household dysfunction variables (battered mother, household substance misuse, mental illness or depression in household, parental separation or divorce, incarcerated household member). For each case, all ACEs were coded as “no” or “yes,” based on the total available interview material.

Stressful life events in the previous year were scored following a modified non-adult version of the Holmes and Rahe Social Readjustment Rating Scale [SRRS; (54)], based on the Adolescent Life Change Event Scale (55). To the original 39 non-adult items we added the following age-relevant items: imprisonment, exposed to violence, moving away from home, increase in arguments with parents or partner, economic difficulties, and starting or interrupting work or studies. Each of the 45 items was ascribed a Life Change Unit (LCU) on a 100-point scale (56). The Social Readjustment Index (SRI) is the sum of all LCU scores. Following a study of SRRS as a suicide risk scale (30), we also calculated the Life Event Index (LEI), i.e., the total number of stressful life events the previous year for each case.

The interview protocol included the 24-item Shortened Ways of Coping Questionnaire (WCQ), one of the most frequently used coping scales. The answers were binary (yes-no) rather than on a scale, and the questions were not situation-specific. The original Ways of Coping Checklist (57) contained 68 items that described coping options the subject indicated were or were not used in specific stressful situations. Following a factor analysis, eight scales were created (58–60): Confrontive Coping, Distancing, Self-Controlling, Seeking Social Support, Accepting Responsibility, Escape-Avoidance, Planful Problem-Solving, and Positive Reappraisal. There are now several versions of the WCQ with varying numbers of items and different subscales. For example, factor analysis of the Canadian 24-item version (61) revealed four factors, whereas factor analysis of the French 27-item version (62) gave three dimensions.

### Statistical Analyses

In order not to analyze all 24 dichotomous WCQ items, and because previous studies have indicated differing number of dimensions, we performed factor analysis with oblimin rotation of the 229 responses. Four dimensions were identified. As a next step, factor scores were calculated for each case. An ANOVA and Tukey's honestly significant difference (HSD) *post-hoc* test were used for testing between-group differences in coping.

**Figure 1:**
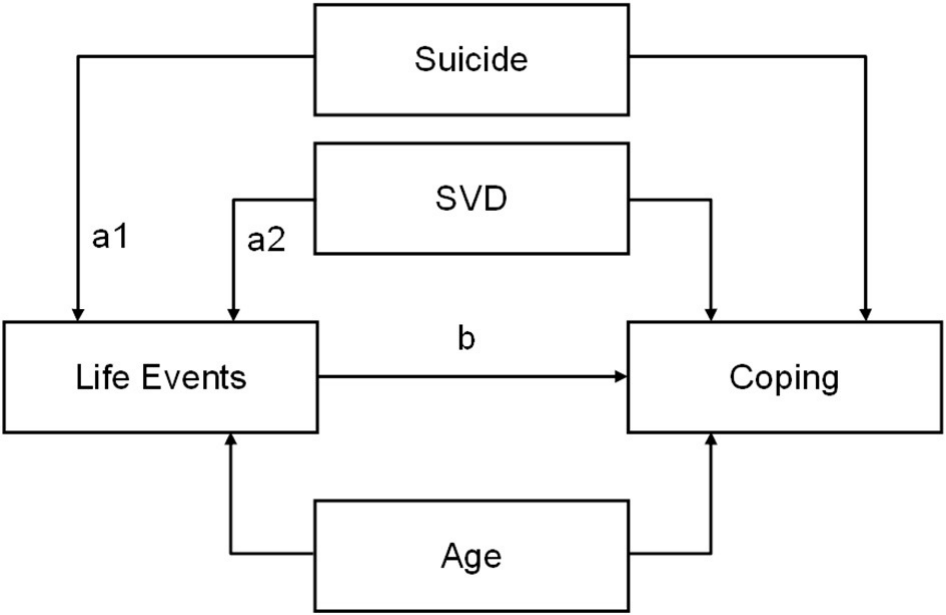
Illustration of the conducted mediation analyses with the control group as reference. For suicide vs. control the size of the indirect/mediated effect equals a1 × b and for sudden violent death (SVD) vs. control a2 × b.

In order to investigate how between-group differences in coping were accounted for by differences in negative life events, early and late in life, 12 separate mediation analyses were conducted with membership in the three groups as the independent variable, one of the four coping strategies as the dependent variable, and one of the three life events measures as a mediator (Figure 1). The effects were adjusted for age and studied for the full sample as well as separately for males and females. The aim was to evaluate whether life events could, to some degree, account for differences in coping strategies between the three groups, i.e., we did not intend to make any causal claims. The standard error and *p*-value of the mediated effects were calculated through 5,000 bootstrapped subsamples.

This paper includes 73 significance tests, which increases the risk for false positives above the nominal 5%. With a strict Bonferroni correction the significance level would be set at 0.05/73 = 0.0007. However, this would, of course, increase the risk for false negatives [for the critique of excessive claims for statistical significance and dismissal of possibly crucial effects, see (63)].

## Results

Factor analysis of the WCQ responses in 229 cases indicated a four-factor solution, explaining 54.2% of the variance (Table 1). Two of these factors can be regarded as more adaptive coping: (1) Planful Problem-Solving and (3) Seeking Social Support, whereas two further factors represent more maladaptive coping: (2) Escape-Avoidance and (4) Confrontive Coping (aggressive, hostile, acting-out).

**Table 1:** Factor analysis of ways of coping with oblimin rotation (factor loadings, eigenvalue and percentage of explored variance).

**Ways of coping items**	**Factor 1**	**Factor 2**	**Factor 3**	**Factor 4**
**1. Planful problem-solving**				
Tried to see the situation as an opportunity to develop	**0.836**	0.007	0.111	−0.078
Was inspired to do something creative about the problem	**0.756**	0.055	−0.088	0.068
Changed or grew as a person in a good way	**0.741**	−0.231	−0.059	−0.030
Tried to see things from the other person's point of view	**0.722**	0.133	0.143	−0.164
Spend more time and energy to make things work	**0.714**	−0.110	0.084	0.150
Tried to organize and plan his/her life better	**0.689**	0.149	0.416	−0.031
Came up with a couple of different solutions to the problem	**0.675**	−0.176	−0.135	−0.145
Made light of the situation; refused to get too serious about it	**0.618**	0.280	−0.378	0.164
Tried to obtain information in order to deal with the problems in an objective manner	**0.587**	−0.312	−0.038	0.312
Took it easy	**0.492**	0.003	−0.272	−0.476
Bargained or compromised to get something positive from the situation	**0.401**	−0.203	−0.047	0.374
**2. Escape-avoidance**				
Kept others from knowing how bad things were	−0.062	**0.772**	−0.096	0.107
Got bad conscience, did not want to bother others, complained not the problems, kept others from knowing how bad things were	0.071	**0.731**	0.054	0.013
Got away from it for a while; tried to rest or take a vacation	0.141	**0.693**	0.169	0.017
Waited and hoped the problems would resolve themselves	−0.230	**0.638**	0.056	−0.216
Tried to make him-/herself feel better by eating, drinking and smoking	−0.237	**0.473**	0.398	0.242
**3. Seeking social support**				
Tried to analyze the problem in order to understand it better	0.130	0.083	**0.689**	−0.035
Get anxious	−0.065	0.262	**0.631**	0.157
Looked for support and help	0.228	−0.436	**0.547**	−0.045
Asked a relative or friend he/she respected for advice	−0.036	−0.551	**0.539**	−0.008
Prayed	0.041	0.221	**0.343**	0.001
**4. Confrontive coping (aggressive-acting-out)**				
Took a big chance or did something very risky	0.069	0.102	−0.109	**0.818**
Expressed anger to the person(s) who caused the problem	0.000	−0.205	0.082	**0.665**
Tried to make him-/herself feel better by using drugs or medication	−0.323	0.395	0.203	**0.467**
**Eigenvalue**	5.234	3.424	2.305	2.048
**Percentage of explained variance**	0.218	0.143	0.096	0.085
**Percentage of explained variance, cumulative**	0.218	0.361	0.457	0.542

*The hihgest factor loadings for items composing each of the four factors are in bold and shaded*.

Correlations among age, life events and ways of coping for all cases and groups are presented in Table 2. Planful Problem-Solving correlated negatively with Escape-Avoidance and Confrontive Coping, whereas Seeking Social Support was positively correlated with Confrontive Coping. The two maladaptive coping strategies were positively correlated with each other. We found no significant correlation between age and life events (ACE, SRI, LEI) and a weak positive correlation between age and both Planful Problem-Solving and Confrontive Coping. SRI and LEI correlated almost perfectly with each other, indicating a strong relation between the severity and the number of recent stressful life events. ACE, SRI, and LEI correlated negatively with Planful Problem-Solving and positively with maladaptive coping (Escape-Avoidance and Confrontive Coping). This general picture seemed to hold both for men and for women.

**Table 2:** Correlations between age, life events, and coping strategies (Pearson's *R*).

	**2. ACE**	**3. SRI**	**4. LEI**	**5. Planful**	**6. Support**	**7. Escape**	**8. Confrontive**
1. Age	0.129	0.060	0.095	0.164^*^	0.066	0.064	0.182^**^
2. ACE		0.319^***^	0.315^***^	−0.287^***^	0.119	0.401^***^	0.250^***^
3. SRI			0.979^***^	−0.238^***^	0.100	0.392^***^	0.283^***^
4. LEI				−0.259^***^	0.109	0.392^***^	0.289^***^
5. Planful problem-solving					−0.087	−0.316^***^	−0.136^*^
6. Seeking social support						0.048	0.152^*^
7. Escape-avoidance							0.185^**^
8. Confrontive coping							

*** *p < 0.001*,

** *p < 0.01*,

* *p < 0.05*.

**Figure 2:**
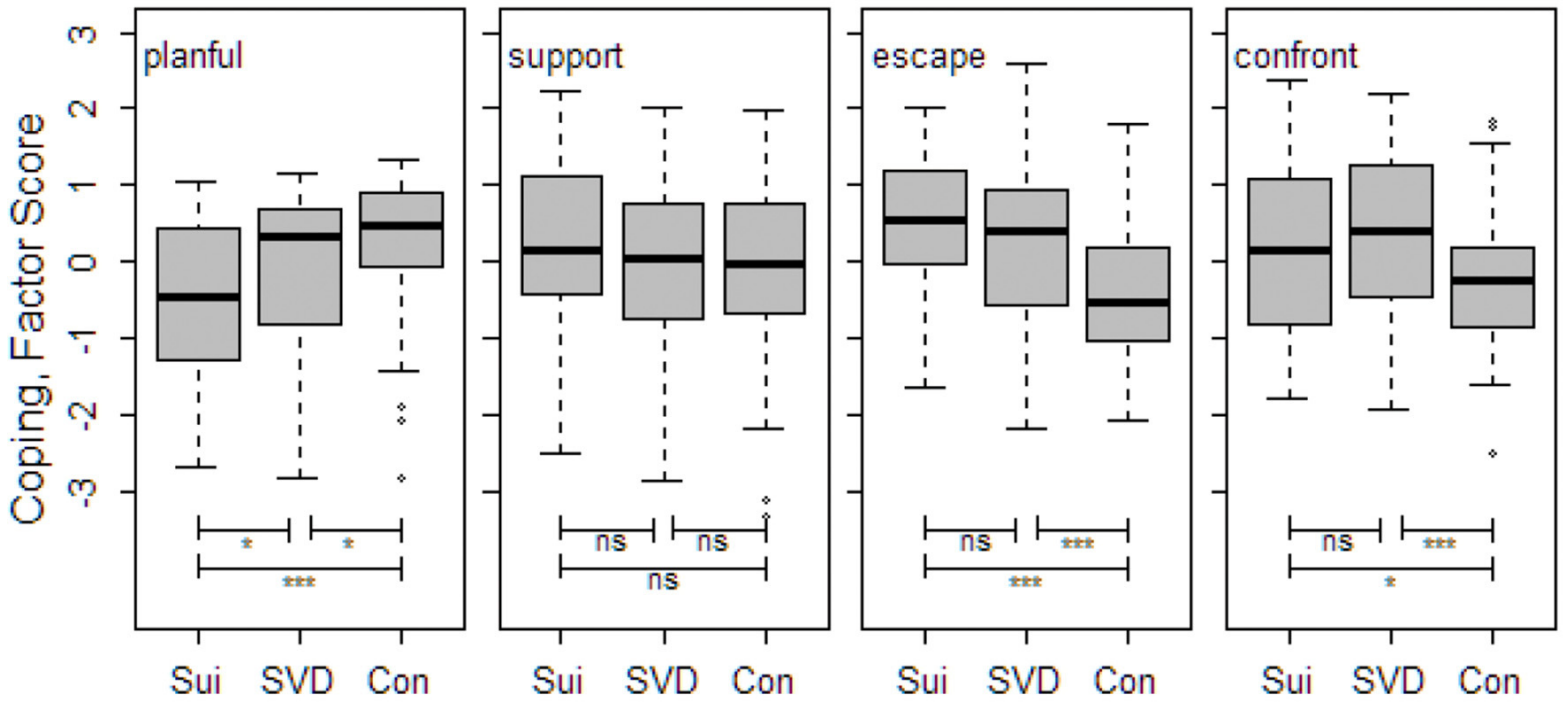
Between-group differences in coping styles (median factor score, interquartile range, and range) for the four factors, and significance levels. ***p < 0.001, *p < 0.05, ns, non-significant; Sui, suicide group; SVD, sudden violent death group; Con, control group.

Between-group differences in coping strategies are presented in Figure 2. Table 3 shows mean scores on the coping factors and measures of life events, as well as *p*-levels for between-group differences for all subjects and for males and females separately. Planful Problem-Solving had the highest level in the control group and the lowest level in the suicide group, with significant differences between all three groups. Surprisingly, there were no significant between-group differences in Seeking Social Support. Escape-Avoidance had the highest level in the suicide group, followed by SVD, and the lowest level in the control group, with significant differences between suicide and controls, and SVD and controls. Confrontive Coping had the highest level in the SVD group, followed by suicides, and the lowest level in the control group, with significant differences between suicide and controls and between SVD and controls. Women in the suicide group had higher scores for both Seeking Social Support and Confrontive Coping than men. In the SVD group, women had higher scores for Planful Problem-Solving than men but lower scores for Seeking Social Support, Escape-Avoidance and Confrontive Coping. As a reference, women in the control group had lower scores for Planful Problem-Solving than men, but higher scores for both Seeking Social Support and Escape-Avoidance.

**Table 3:** Mean scores (and SD) on the four coping factors as well as three measures of life events, separately for the three groups.

	**Suicide**		**Sudden violent death**		**Controls**	
	***M*** **(SD)**		***M*** **(SD)**		***M*** **(SD)**	
**Coping strategies**						
Planful problem-solving (factor 1)	−0.593 (1.26)		−0.053 (1.286)		0.407 (0.948)	
Seeking social support (factor 3)	0.203 (1.152)		−0.103 (1.186)		−0.078 (1.138)	
Escape-avoidance (factor 2)	0.613 (0.978)		0.199 (1.283)		−0.495 (1.009)	
Confrontive coping (factor 4)	0.187 (1.222)		0.337 (1.268)		−0.334 (0.882)	
**Life events**						
ACE	2.16 (1.55)		1.82 (1.52)		1.13 (1.01)	
SRI	148.14 (56.32)		111.23 (58.87)		76.25 (62.32)	
LEI	2.84 (1.05)		2.1 (1.07)		1.41 (1.13)	
	**Males**	**Females**	**Males**	**Females**	**Males**	**Females**
**Coping strategies**						
Planful problem-solving (factor 1)	−0.47 (1.07)	−0.62 (1.05)	−0.10 (1.11)	0.12 (0.80)	0.40 (0.67)	0.06 (1.03)
Seeking social support (factor 3)	0.02 (1.04)	0.48 (0.86)	−0.03 (0.96)	−0.38 (1.51)	−0.28 (1.00)	0.57 (0.59)
Escape-avoidance (factor 2)	0.57 (0.86)	0.50 (0.79)	0.25 (1.10)	−0.25 (0.97)	−0.49 (0.85)	−0.13 (0.85)
Confrontive coping (factor 4)	−0.06 (1.02)	0.60 (1.10)	0.36 (1.12)	0.02 (1.20)	−0.28 (0.81)	−0.26 (0.73)
**Life events**						
ACE	1.98 (1.46)	2.50 (1.68)	1.85 (1.54)	1.57 (1.40)	1.05 (1.00)	1.32 (1.02)
SRI	146.2 (55.89)	151.77 (58.27)	109.29 (59.50)	126.43 (55.33)	73.49 (61.56)	83.75 (64.89)
LEI	2.78 (1.06)	2.95 (1.05)	2.04 (1.07)	2.57 (0.98)	1.37 (1.13)	1.54 (1.14)
**Tukey HSD** ***p*** **-values for between-group differences**	**Suicide vs. controls**		**SVD vs. controls**		**Suicide vs. SVD**	
Planful problem-solving (factor 1)	0.000		0.035		0.032	
Seeking social support (factor 3)	0.316		0.991		0.340	
Escape-avoidance (factor 2)	0.000		0.000		0.107	
Confrontive coping (factor 4)	0.013		0.001		0.743	
ACE	0.000		0.003		0.331	
SRI	0.000		0.001		0.002	
LEI	0.000		0.000		0.001	

As shown in Table 3, there were significant differences between both the suicide and the SVD groups vs. the controls for all measures of life events, and between the suicide and the SVD groups for recent stressful life events (SRI and LEI), but not for ACEs. Furthermore, we found higher levels of stressful life events among females than males for all measures and in all groups, except for ACEs in the SVD group.

As we found several significant between-group differences in coping strategies and life events, in the next step we investigated the potentially mediating role of early and late life events, i.e., whether life events could account for differences in coping to any degree. Indirect/mediated effects of group membership on coping *via* life events, adjusted for age, are presented in Table 4. By dividing these values with the corresponding total effects (corresponding to the difference between the group means when adjusting for age) we get estimations of how much of the difference in coping between groups is accounted for by differences in life events. The differences in Escape-Avoidance between the suicide and the SVD groups, compared to the controls, are to some degree accounted for by differences in ACEs (approximately 24% for suicide and 26% for SVD, respectively), SRI (31 and 24%), and LEI (32 and 25%). Furthermore, differences in ACEs can account for some of the differences in Planful Problem-Solving (20 and 32%), whereas differences in SRI (51 and 19%) and LEI (55 and 20%) can to some degree account for the differences in Confrontive Coping. As can be seen in Table 4, the mediated effects are quite similar in the suicide and the SVD group.

**Table 4:** Mediated/indirect effects (and standard errors), adjusted for age, of group membership (suicide and sudden violent death vs. controls) on coping *via* life events (ACE, SRI, and LEI).

	**Suicide**			**Sudden violent death**		
**Coping strategies**	**ACE**	**SRI**	**LEI**	**ACE**	**SRI**	**LEI**
Planful problem-solving	−0.171 (0.062)^**^	−0.136 (0.079)	−0.173 (0.086)^*^	−0.120 (0.052)^*^	−0.066 (0.042)	−0.083 (0.045)
Seeking social support	0.072 (0.055)	0.070 (0.089)	0.086 (0.100)	0.049 (0.039)	0.036 (0.047)	0.043 (0.052)
Escape-avoidance	0.224 (0.069)^***^	0.292 (0.087)^***^	0.303 (0.093)^***^	0.153 (0.053)^**^	0.145 (0.058)^*^	0.148 (0.06)^*^
Confrontive coping	0.125 (0.065)	0.236 (0.085)^**^	0.254 (0.090)^**^	0.086 (0.048)	0.117 (0.054)^*^	0.125 (0.056)^*^

*** *p < 0.001*,

** *p < 0.01*,

* *p < 0.05*.

*The values correspond to the a1/a2 × b product in Figure 1. For example, solely due to the difference in ACE between the suicide and control groups, the former are expected to have a score on Planful Problem-Solving that is 0.171 standard deviations lower compared to the latter (controlling for age)*.

## Discussion

The four coping strategies identified in the present study are also represented among the original eight scales of the WCQ (58, 59). Between-group comparisons showed that distinctive for both the suicide and the SVD group was significantly less Planful Problem-Solving, more Escape-Avoidant, and more Confrontive Coping than among the controls. Furthermore, the suicide group had the highest level of Escape-Avoidance and the SVD group the highest level of Confrontive Coping. In previous research, two different kinds of coping with adversities and strains in life are conceptually linked to internalizing disorders (depression, anxiety disorder, phobic, panic, and obsessive-compulsive) and externalizing disorders (antisocial personality, substance dependence), both playing a part in suicidal behavior (17, 41, 43, 64, 65). Of the two maladaptive coping strategies represented in the present study, Escape-Avoidance corresponds to internalizing, and Confrontive Coping to externalizing ways of coping. These results might confirm the suggestion from our previous study (47) that common to the suicide and the SVD groups is a mix of internalizing and externalizing psychopathology and coping, whereas the SVD group is distinguished by mostly externalizing psychopathology and coping strategies. A new finding was that the suicide group had significantly less Planful Problem-Solving even in comparison with the SVD group.

Furthermore, both groups experienced significantly more adverse childhood experiences and recent stressful life events than the controls—the suicide group being more exposed to recent stressful life events even in comparison with the SVD group. This might indicate that ACEs are a risk factor for both causes of death, whereas proximal stressful life events are a risk factor for death by suicide to a higher degree than for SVD.

Mediator analyses showed that differences between both of the target groups and controls in Escape-Avoidance were partly accounted for by differences in negative life events, early and late in life, and differences in Confrontive Coping were accounted for by differences in recent stressful life experiences, whereas differences in Planful Problem-Solving were accounted for by differences in adverse childhood experiences. The mediated effects were quite similar in the two target groups.

Taken together, our results are congruent with ACE studies showing a powerful relationship between ACEs and risk of attempted suicide through the life span (66). Furthermore, childhood trauma exposure has been linked to post-traumatic risk-seeking and justice involvement in adolescence (26). In the general population of adolescents and young adults, more adaptive coping has a protective function in regulating stress and is related to well-being (67). Accordingly, we found Planful Problem-Solving to be significantly more represented in the control group than in both of the target groups. In the control group, we found that ACEs were associated with less Planful Problem-Solving. Moreover, our study confirmed previous findings (14) suggesting that stressful life events to some degree accounted for the differences in coping between the controls and both the suicide and the SVD group.

We found a weak positive correlation between age and both Planful Problem-Solving and Confrontive Coping, suggesting an association between maturational processes in adolescence and emerging adulthood, and both adaptive coping and maladaptive externalizing coping.

The patterns of gender differences in coping strategies are more difficult to interpret, due to the low power in gender-specific subgroups. We found that women generally were more exposed to stressful life events than men in all three groups, with the highest levels of both early and proximal stressful life events in the suicide group. These results seem to indicate that the differences in associations between life events and coping strategies require further studies not only among the suicide group, the SVD group and the controls, but also between genders within the groups.

### Implications for Prevention and Intervention

Surprisingly, on the general level, we found no significant between-group differences in Seeking Social Support. Empirical studies have demonstrated that the majority of people with suicidality do not seek help (68). Studies of adolescents and young adults (69, 70) have suggested several barriers to help-seeking, such as stigmatization, fear of confidentiality being breached, and high self-reliance. In a Canadian study of children and youths who died by suicide (71), most of the subjects had been seen by an outpatient physician and in a hospital emergency department in the previous year, but not all received mental health care. A systematic review (68) found that suicidality and mental health problems generally decrease help-seeking for perceived suicidal ideation while increasing actual service use. A previous qualitative study of the parents' perspective on youth suicide (72) found that the young persons and their parents typically asked for professional help, but did not receive the help they needed. Thus, barriers against help-seeking are not only related to the young persons themselves but include professionals and the organization of routine mental health care. Clinical enquiries should include, besides suicidal thoughts, also ACEs, recent life stressors, and coping.

In our study, 71% of those who died by suicide had had some form of contact with psychiatric care. Regular follow-up of such contacts may be decisive for preventing death by suicide. Frequent outpatient psychiatric contacts and pharmacotherapy combined with psychotherapy have been demonstrated to be associated with decreased risk for suicide in psychiatric patients (73). On the other hand, 42% of the SVD group had no psychiatric contact. Even if the difference is hardly significant, this might indicate that the use of externalizing, aggressive, hostile, risk-taking coping can constitute in itself a barrier against help-seeking. Consequently, other kinds of interventions are needed in social services, youth organizations, schools, etc., focusing on signs of antisocial behavior and maladaptive coping. Unintentional injury is the most common cause of death among young people both in the US and Europe (38, 74, 75). Counteracting life-threatening behavior among young people, hostile contempt, violence in interpersonal and intergroup relationships, and externalization and projection onto others of own shortcomings and weaknesses, is one of the great challenges of our time.

Furthermore, social interventions focusing on prevention of adverse childhood experiences and adequate treatment of persons affected by them might lead to improved suicide prevention [cf. (66)], as well as prevention of sudden violent death. In childhood, adolescence and emerging adulthood, even minor shortcomings might be experienced as catastrophic. Our study showed that recent strains in life were associated with Confrontive Coping, both in the suicide and SVD groups. Maladaptive externalizing coping can result in new strains in life, thus creating a vicious circle of life events and coping with lethal outcomes. There is no effective algorithm to predict suicide [cf. (20)] and other forms of sudden violent death in each particular case. However, improved recognition and understanding of the interplay between life events, both in the distant past and the present, and coping styles, besides other well-known risk factors, may facilitate the identification of young people at risk of suicide and other violent death.

### Strengths and Limitations

As far as we are aware, no previous study has explored the interactions between life events and coping strategies, comparing cases of suicide and sudden violent death. Inclusion of living matched controls from the general population strengthens the credibility of between-group comparisons. The collection of consecutive medico-legal cases with relatively low attrition contributes to the representativity and generalizability of our results. However, the psychological autopsy method, although providing valuable and not otherwise accessible knowledge, entails methodological concerns (37). The interview responses are inevitably adjusted to the already known outcome. “Search after meaning” might result in informants identifying a number of internal or external factors that could explain the death (5). In the control group, not only relatives but also living young people were included, thus resulting in what Brent (4) called “asymmetry of informants.” Social desirability bias might influence answers in all three groups, resulting in underreporting of adverse life events. Psychological autopsy studies have to be accompanied by more expensive and labor-intensive longitudinal research, enabling us to study these interactions across time, i.e., as processes, and including the issues of age and gender differences.

The most important limitation of this study is the large number of comparisons, thus increasing the risk for type I error. Accordingly, the results must be interpreted with caution. Considering the small number of females included and, consequently, low power in gender comparisons, we limited ourselves to presentation of gender differences on the descriptive level of mean values. Hopefully, future studies with larger samples and higher power can shed more light on the interesting and potentially important issue of gender differences. A further limitation is that the measures we have used for distal and proximal life stressors and coping styles are probably not specific enough to catch the psychologically important features of suicide and other forms of sudden violent death among young persons. Other risk factors, studied in our previous publication (47), together with both life stressors and inadequate coping, might be decisive in each individual case. Both suicide and other forms of sudden death among young people are extremely infrequent, and therefore potentially more exceptional than what can be predicted by standard measurements and scales for ways coping and life stressors. Accordingly, case studies and qualitative methods, as well as multimethod studies, can contribute in a substantial way to extending our knowledge beyond these limitations [cf. (72)].

### Conclusions

Both the suicide and the SVD group had significantly less Planful Problem-Solving, more Escape-Avoidant, and more Confrontive Coping than the controls. The highest level of Escape-Avoidance and the lowest level of Planful Problem-Solving were found in the suicide group, whereas the SVD group had the highest level of Confrontive Coping. Both groups were exposed to significantly more adverse childhood experiences and recent stressful life events than the controls; however, the suicide group experienced more recent stressful life events even in comparison with the SVD group. Differences between the suicide and the SVD groups vs. controls in Escape-Avoidance were partly accounted for by both adverse childhood experiences and recent stressful life events. Differences in Confrontive Coping were partially accounted for by recent stressful life events, whereas differences in Planful Problem-Solving were partially accounted for by adverse childhood experiences. These results might have important implications for prevention and treatment of both suicide and other forms of life-threatening behavior.

## Data Availability Statement

The raw data supporting the conclusions of this article will be made available by the authors, without undue reservation.

## Ethics Statement

The studies involving human participants were reviewed and approved by the Regional Ethical Review Board, Karolinska Institutet, Stockholm (Reference Number 96:204 and 2005/530-32). Written informed consent to participate in this study was provided by the participants' legal guardian/next of kin. Written informed consent was obtained from the individual(s), and minor(s)' legal guardian/next of kin, for the publication of any potentially identifiable data included in this article.

## Author Contributions

AWT performed the data collection, designed the present study, and wrote and revised the manuscript. KS performed the statistical analyses, contributed substantially to the interpretation and presentation of the results. BR and P-AR participated in planning the study, interpretation of data, and revised the manuscript critically. All authors have read and approved the final manuscript.

## Conflict of Interest

The authors declare that the research was conducted in the absence of any commercial or financial relationships that could be construed as a potential conflict of interest.

## Publisher's Note

All claims expressed in this article are solely those of the authors and do not necessarily represent those of their affiliated organizations, or those of the publisher, the editors and the reviewers. Any product that may be evaluated in this article, or claim that may be made by its manufacturer, is not guaranteed or endorsed by the publisher.
